# Effect of Number of Touches and Exercise Duration on the Kinematic Profile and Heart Rate Response During Small-Sided Games in Soccer

**DOI:** 10.2478/hukin-2014-0039

**Published:** 2014-07-08

**Authors:** David Casamichana, Luis Suarez-Arrones, Julen Castellano, Jaime San Román-Quintana

**Affiliations:** 1 Faculty of Physiotherapy and Speech Therapy Gimbernat-Cantabria. University School associated with the University of Cantabria (EU Gimbernat-Cantabria). Torrelavega, Spain.; 2 MasterdeFutbol. Pablo de Olavide University. Seville. Spain.; 3 Faculty of Physical Activity and Sport Sciences. University of the Basque Country (UPV/EHU). Vitoria-Gasteiz, Spain.

**Keywords:** soccer, bout duration, exercise intensity, small sided games, time-motion, GPS device, heart rate

## Abstract

This study aimed to examine the effect of exercise duration and the number of touches allowed during possession on time-motion characteristics and the physiological responses of soccer players in 6 vs. 6 small-sided games (SSGs) lasting 12 minutes. The analysis divided each game into two 6-min periods and we compared two formats: free play (SSG_FP_) vs. a maximum of two touches per individual possession (SSG_2T_). Participants were 12 semi-professional players (age: 22.7±4.3 years; body height: 177.5±4.9 cm; body mass: 74.9±6.3 kg) and the following variables were measured by means of heart rate monitors and GPS devices: mean heart rate (HRmean), time spent in each exercise intensity zone, total distance covered, total distance covered in different speed zones, number of accelerations at different intensities, maximum speed reached, player load, and the work-to-rest ratio. The results showed that in SSGFP there was a decrease in the intensity of physical parameters during the second 6-min period (6–12 min), whereas this decrease was not observed when a maximum of two touches per individual possession was allowed. During the second period (6–12 min) of SSG2T there was an increase in HRmean and in the time spent in high exercise intensity zones, but these differences were not observed in SSGFP. The value of these findings for soccer coaches is that they illustrate how different technical, tactical or conditioning objectives could be addressed by altering the length and format of the SSG used in training.

## Introduction

Small-sided games (SSGs) are widely used as part of training (Aguiar et al., 2012) in several sports such as soccer (Hill-Haas, Dawson et al., 2011; Köklü et al., 2012) or rugby (Gabbett et al., 2012; Kennett et al., 2012) and they enable coaches to address various objectives simultaneously, for example, technical skills (Jones and Drust, 2007), conditioning (Hill-Haas, Dawson et al., 2009), tactical behavior (Almeida et al., 2013; Sampaio and Maçãs, 2012), or psychological aspects (Flanagan and Merrick, 2002). If training is to be implemented systematically, the different elements involved need to be taken into consideration when designing drills, as these elements can affect the demands placed on players (Fanchini et al., 2011; Tessitore et al., 2006). One key element is the duration of a training drill, as this influences both the amount and intensity of training demands (Fanchini et al., 2011). Research to date has considered a wide range of bout durations when studying the effect of training drills, from as little as 1.5 minutes (Aroso et al., 2004; Dellal et al., 2008) up to 24 minutes (Hill-Haas, Coutts et al., 2010).

A further aspect to be considered is the duration and number of repetitions. Increasing the duration could influence player’s behavior by encouraging greater self-regulation and reducing exercise intensity. Intensity may also decrease as a result of fatigue (Fanchini et al., 2011). As for the number of repetitions, several different proposals have been reported in the context of SSGs, ranging from 25 (Rodrigues et al., 2007) down to 10 (Katis and Kellis, 2010) or even a single bout (Hill-Haas, Coutts et al., 2010). However, the most common format involves between three and six repetitions (Hill-Haas et al., 2011).

It is difficult to compare the effects of the temporal dimension across studies that differ not only in the duration of bouts but also in the number of repetitions and other related variables (number of players per side, pitch size, use of goals, among others), not least as these aspects sometimes vary within the same study (Koklü et al., 2011). In fact, very few studies have specifically examined the effect of the temporal variable on player’s response. Tessitore et al. (2006) studied exercise intensity in 6 *vs.* 6 drills of different durations (3 and 8 min) and found that during 8-min drills, players spent more time above the anaerobic threshold. More recently, Fanchini et al. (2011) sought to determine whether an increase in the duration of repetitions (2, 4, and 6 min) affected exercise intensity and technical performance during 3 *vs.* 3 SSGs. They found that the heart rate was significantly lower during the 6-min bout compared with the 4-min bout (87.8 ± 2.8% HR_max_ *vs.* 88.5 ± 3.1% HR_max_), although there were no significant differences in terms of technical performance. The authors concluded that an increase in duration could lead to a decrease in intensity, although the difference was unlikely to be of sufficient magnitude to induce different training adaptations.

Despite the relevance of this research, those studies which have considered the temporal dimension of training have not specifically examined its effect on the physical response of players. Consequently, occurrence of the acute effects of exercise duration in player’s performance is not clear. In light of this, the aims of the present study were as follows: 1) to analyze the physical and physiological response of players across 12-min SSGs that were divided into two 6-min periods; and 2) to examine this temporal influence in two different SSGs: free play (SSG_FP_) versus a maximum of two touches per individual possession (SSG_2T_).

## Material and Methods

### Sample

Participants were 12 semi-professional male soccer players (age: 22.7 ± 4.3 years; body height: 177.5 ± 4.9 cm; body mass: 74.9 ± 6.3 kg; YYIRT1: 2360 ± 638.4 m) who played for the same team (senior division). They had played federation soccer for a mean of 12.5 years prior to the study. Their standard training involved three or four sessions per week (each lasting around 90 min) in addition to a competitive match. All the players were notified of the research design and its requirements, as well as the potential benefits and risks, and they gave their informed consent prior to the start. The Ethics Committee of the University of the Basque Country (CEISH) gave its institutional approval of the study.

### Procedure

The study was conducted over a three-week period (January, under similar weather conditions) of the 2010–11 competitive season. Players were familiarized previously with the two types of SSG and the devices that would be used during the study. One week before the experimental part of the study commenced, players simultaneously performed the Yo-Yo Intermittent Recovery Test-Level 1 (YYIRT1) in order to determine their HR_max_. This was done on an outdoor artificial pitch with the players wearing soccer cleats.

Four training sessions (with an interval of at least 48 h between them) were subsequently conducted on an outdoor artificial grass pitch and at similar times of the day (8:30 p.m.) in order to avoid circadian effects on performance (Drust et al., 2005). Each session began with a 15-min standard warm-up followed by a single drill lasting 12 min. This drill was a SSG involving the same number of players per side (6 *vs.* 6 without goalkeepers) and played in two different ways: sessions 1 and 3 involved free play whereas in sessions 2 and 4 each player was allowed a maximum of two touches per possession.

In order to avoid potential imbalances between the two teams and to ensure their equivalence, we followed the procedure proposed by Casamichana and Castellano (2010), whereby players were classified according to the following variables: minutes of competitive play, performance on the YYIRT1, usual playing position and subjective appraisal of the coach.

To avoid the pacing effect (Sampaio et al., 2013), coaches were present during all the SSGs in order to offer encouragement to the players (Rampinini et al., 2007). In addition, eight footballs were distributed around the edge of the pitch in order to maximize the effective playing time (Casamichana and Castellano, 2010). All participants were advised to maintain their normal diet with special emphasis being placed on a high intake of water and carbohydrates.

### Independent Variables

The independent variables were the two 6-min periods (0.0–5.9 min and 6.0–12.0 min) within each 6 *vs.* 6 SSG. The aim of the players during these 12-min SSGs was simply to maintain possession for as long as possible. Two types of game with possession rules were used: free play *vs.* a maximum of two touches per individual possession. The pitch size was the same in all the SSGs (60 × 49 m), such that the relative area per player was 245 m^2^. Except for the offside rule, the standard rules of 11-a-side soccer were followed.

### Physiological Profile: Heart Rate

The physiological profile was assessed on the basis of the heart rate (Esposito et al., 2004), which was recorded every 5 s using a telemetric device (Polar Team Sport System, Polar Electro Oy, Finland). Exercise intensity was expressed in relation to the individual maximal heart rate (HR_max_) obtained during the YYIRT1 (Bangsbo et al., 2008). HR data were classified based on the percentage of total time spent in each of the following four HR zones: <80%HR_max_, 81–90%HR_max_, 91–100%HR_max_, and >80%HR_max_ (the latter accumulates all values above 80% of HR_max_). Data were categorized into HR zones using the software Logan Plus 4.5.0 (Catapult Innovations, 2010).

### Time-Motion Characteristics: Distance Covered and Number of Accelerations Performed

The running profile was measured using a portable global positioning system (GPS) device operating at a sampling frequency of 10 Hz (MinimaxX 4.0, Catapult Innovations). After recording, the data were downloaded to a PC and analyzed using Logan Plus 4.5.0 (Catapult Innovations, 2010). Similarly to previous studies, four speed zones (0.1–6.9, 7.0–12.9, 13.0–17.9, and ≥18.0 km·h^−1^) (Hill-Haas, Dawson et al., 2009; Impellizzeri et al., 2006) and four acceleration zones (1.0–1.4, 1.5–1.9, 2.0–2.4, and ≥2.5 m·s^−2^) (Varley et al., 2012) were established. The total distance covered (TD), maximum speed reached (V*_max_*), work-to-rest ratio, player load, distance covered within designated speed zones and the number of accelerations within designated zones were all calculated. Player Load (PL) is a measure calculated using the data obtained via the triaxial accelerometer incorporated within the GPS device (Boyd et al., 2011; Casamichana et al., 2013; Gastin et al., 2013). PL has demonstrated high reliability, both within and between devices, thereby suggesting that accelerometers are a viable tool for tracking activity changes during exercise (Akenhead et al., 2013; Varley et al., 2012). PL was calculated using the following formula and expressed per minute of practice (AU•min^−1^):

$$\begin{array}{l} \text{Player}\;\text{load}=\surd ((({\text{aca}}_{\text{t}}=\text{i}+1-{\text{aca}}_{\text{t}}=1)2+\\ \left({\text{act}}_{\text{t}}=\text{i}+1-{\text{act}}_{\text{t}}=1)2+({\text{acv}}_{\text{t}}=\text{i}+1-{\text{acv}}_{\text{t}}=1)2)/100\right) \end{array}$$

where *aca* is the acceleration in the anteroposterior or horizontal axis, *act* is the acceleration in the transverse or lateral axis, *acv* is the acceleration in the vertical axis, *i* is the current time, and *t* is time.

The technology used to collect these data has been previously validated and shown to be reliable for monitoring high intensity activities in soccer players (Castellano et al., 2011; Varley et al., 2012).

### Statistical analysis

Variables are presented as mean (±SD). The precision of estimates is indicated with 90% confidence limits. In addition to the analyses (i.e., paired t tests) conducted to identify any statistically significant differences in movement patterns and heart rate responses during both types of SSG, pairwise comparisons were analyzed for practical significance using magnitude-based inferences (Hopkins, 2006). The movement patterns and heart rate data were log-transformed prior to analysis in order to reduce non-uniformity of error. Intraclass correlation coefficient (ICC) with 95% CI was used to determine between-subject reliability of the SSG. Within-subject variation for the SSG was determined by calculating the coefficient of variation (CV). Standardized differences or effect sizes (ES, 90% confidence interval) in SSG physical responses and probabilities were calculated to establish whether the true (unknown) differences were lower than, similar to, or higher than the smallest worthwhile difference (0.2 multiplied by the between-subject standard deviation, based on the Cohen’s effect size criterion). Quantitative chances of higher or lower differences were evaluated qualitatively as follows (Batterham and Hopkins, 2006; Hopkins et al., 2009): <1% is almost certainly not, <5% is very unlikely, <25% is unlikely/probably not, 25–75% is possibly/possibly not, >75% is likely/probably, >95% is very likely, and >99% is almost certainly. A substantial effect was set at >75%. If the chance of higher or lower differences was >75%, the true difference was assessed as clear (substantial).

## Results

### Kinematic profile

**Figure 1:**
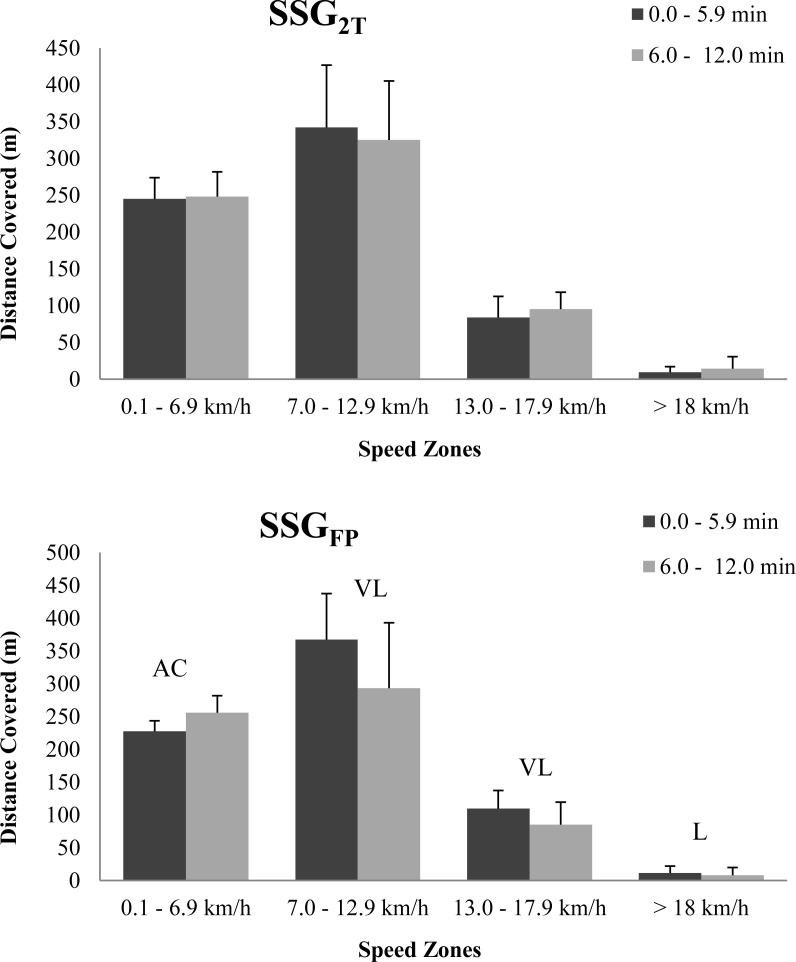
Distance (m) covered within designated speed zones during each 6-min period. SSG_FP_: small-sided game with free play; SSG_2T_: small-sided game with a maximum of two touches per individual possession. L: Likely; VL: Very Likely; AC: Almost Certainly. Data are mean ± SD

Running data for both types of SSG are presented in Table 1 and Figure 1. Test-retest reliability for total distance covered, PL, heart rate, maximum speed and work-to-rest ratio as measured by the CV were 7% (1–32%), 7% (0–24%), 2% (0–11%), 10% 05–31%) and 18% (1–58%), respectively, and the ICC (95% CI) ranged from 0.79 to 0.95. Players’ movement patterns were relatively stable during SSG_2T_ while maximum speed was higher (+6.2%, Table 1) in the second 6-min period of these games.

During the SSG_FP_ a substantial decrease was observed in the second 6-min period in total distance covered (−10.4%, Table 1), distance covered at speeds of 7.0–12.9 km·h^−1^ (−20.2%, Figure 1), 13.0–17.9 km·h^−1^ (−22.7%, Figure 1), ≥18 km·h^−1^ (−32.2%, Figure 1), work:rest ratio (−27.8%, Table 1) and player load (−16.4%, Table 1).

### Heart Rate Responses

**Figure 2:**
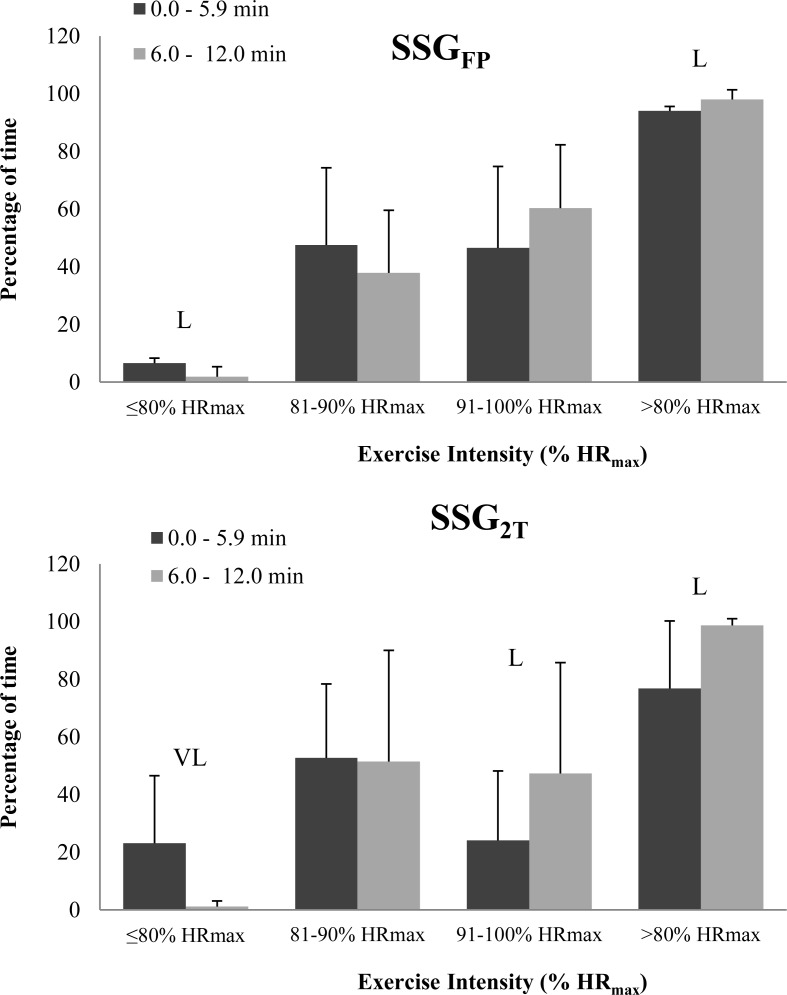
Percentage of playing time spent in each exercise intensity zone during each 6-min period. SSG_FP_: small-sided game with free play; SSG_2T_: small-sided game with a maximum of two touches per individual possession. L: Likely; VL: Very Likely. Data are mean ± SD

During SSG_2T_ the mean heart rate (expressed as a percentage of the HR_max_) increased substantially during the second 6-min period, reaching 89.3 ± 3.1% HR_max_ as compared with 83.8 ± 4.3% HR_max_ in the first 6-min period (+6.2%, *Almost Certainly*). This difference was not reflected between the first and second 6-min periods during SSG_FP_ (89.0 ± 3.1% HR_max_ *vs.* 90.4 ± 2.5% HR_max_, respectively). Figure 2 shows players’ HR responses and the percentage of playing time spent in each HR zone expressed as a percentage of the HR_max_. During the second 6-min period in both types of SSG, players showed a substantial increase in the percentage of time spent at >80% HR_max_ (+22.2% and +4.1%, respectively; Figure 2), although the percentage of time spent at 91–100% HR_max_ only increased substantially in SSG_2T_ (+49%; Figure 2).

## Discussion

This study examined the physiological and physical responses in soccer players across 6 *vs.* 6 SSGs lasting a total of 12 minutes. The aim was to analyze the physical and physiological response across 12-min SSGs and examine the influence of playing time with two different rules: free play versus a maximum of two touches per individual possession. The main findings of the present study were as follows: 1) there was a substantial decrease in running performance during the second 6-min period in SSG_FP_; 2) the mean heart rate and the amount of time players spent in high exercise intensity zone (>90% HR_max_) increased during the second 6-min period during SSG_2T_ but not in SSG_FP_.

Total distance covered during SSG_FP_ decreased substantially during the second 6-min period (6–12 min), due specifically to less distance being covered at speeds ≥7.0 km·h^−1^. These results were similar to those reported by Dellal, Lago-Peñas et al. (2011), although these authors concluded that the observed decrease was likely due to the accumulated number of repetitions, since the duration of each bout was held constant. This decrease in movement patterns was also noted by Casamichana et al. (2013) when comparing SSGs with a total duration of 16 minutes and played in either a continuous or intermittent format. When an intermittent format was used, the total distance covered by players was greater than in case of the continuous game, where a decrease in intensity was observed after the first 4 minutes of play. These results are also consistent with the findings of Hill-Haas et al. (2009) who observed that greater distances were covered at high and sprint speed when the format used was intermittent (4 repetitions × 6 min duration) rather than continuous (24 min duration). The continuous format used in the present study likewise produced a decrease in the intensity of movement variables during the second 6-min period (6–12 min) of the drill, an aspect that needs to be taken into consideration when designing a training schedule.

This is the first study with soccer players that has described the differences between accelerations and decelerations during SSG. Previously, in Australian football (Aughey, 2012) a substantial reduction in the amount of number of maximal accelerations when the competition progressed was observed. Our results during SSG_FP_ also indicated a substantial reduction in PL, the work-to-rest ratio and maximum speed during the second 6-min period (6–12 min), whereas the number of accelerations in the range 2.0–2.4 m·s^−2^ increased slightly during the same period. With regard to exercise intensity, the mean heart rate did not differ between the two 6-min periods, although players did spend substantially longer time at >80%HR_max_ during the second period (6–12 min) what perhaps could be caused by fatigue.

Running performance during SSG_2T_ revealed no differences in movement patterns between the two 6-min periods with the exception of the maximum speed reached, which increased substantially during the second period. Interestingly, the mean heart rate increased during the second 6-min period in the SSG_2T_, there being a substantial reduction in the amount of time players spent at <80%HR_max_. These results were consistent with previous studies that have reported an increase in mean intensity as exercise duration gets longer (Tessitore et al., 2006), being this effect more marked in continuous as opposed to intermittent formats (Hill-Haas, Rowsell et al., 2009). Although it is an aspect that was not considered in the present study, the observed difference may be partly due to the time needed for the heart rate to rise following the start of exercise, which would account for the fact that players spent more time at <80%HR_max_ during the first period. Specifically, it is necessary to take into account the first minute of exercise, this being the time required for players to achieve a high heart rate (Fanchini et al., 2011). A further point to note is that the technical and tactical difficulties produced during the two-touch rule could increase the time which players need in order to adapt to the dynamics of play; indeed, the likelihood of a greater number of ball exchanges could mean that players need longer to achieve a high heart rate. This hypothesis is supported by the fact that players spent a longer time at <80%HR_max_ during the first period in SSG_2T_ than they did during the corresponding period of the SSG_FP_.

The number of touches that individual players are allowed to make in possession of the ball, is a variable that is commonly manipulated by coaches, and it may affect the physical and physiological response of players during SSGs (Aroso et al., 2004; Dellal, Chamari et al., 2011; Dellal, Hill-Haas et al., 2011; Dellal, Lago-Peñas et al., 2011; Sampaio et al., 2007). Previous studies (Dellal et al., 2011; Dellal, Owen et al., 2012) showed that playing with a less number of touches allowed for increased intensity of various parameters (perceived exertion, blood lactate concentration, total distance covered, and distance covered at high intensity). However, in the present study and despite it not being one of the objectives sought, the values of physical load were higher when there was no limit of touches. A possible explanation for this finding, which is contrary to the published data, is that a limit on the number of individual touches may affect players differently depending on their competitive level (Dellal, Hill-Haas et al., 2011), such that those with greater technical and tactical skills, and those who are used to playing with two or fewer touches (Dellal et al., 2010), are better able to adapt to such a limitation. More research is required regarding the extent to which the modification of variables in SSGs may have different effects depending on the motor skill level of the players involved.

The results of the present study showed that the duration of exercise can affect players’ responses, and this highlights the need for the length of drills to be intentionally controlled. If the aim of training is to produce greater cardiovascular output, longer drills should be prescribed. Conversely, if the objective is to encourage high-intensity activity, shorter drills would be more appropriate. Coaches should also take into account that if they decide to limit the number of touches allowed per individual possession, players will take longer to reach 80% of their HR_max_ than would be the case in SSG_FP_.

Changes in any rule during SSGs should be introduced thoughtfully because the effect on physiological and physical responses appears to be influenced by the motor competence of the players involved, by the skill and fluidity with which they are able to perform the drill.

## Figures and Tables

**Table 1:** Running profile and player load for each 6-min period in the two types of small-sided game. Data are mean ± SD

*Variables*	0.0–5.9 min	6.0–12.0 min	ES ± 90% CL	Qualitative Assessment
	SSG_FP_			
Total distance covered (m)	716.3 ± 77.3	642.2 ± 91.1	0.94 ± 0.35	*Almost Certainly*
Maximum speed (km·h^−1^)	18.8 ± 2.2	17.4 ± 2.6	0.63 ± 0.74	*Likely*
Accelerations of 1.0–1.4 m·s^−2^	4.4 ± 2.2	4.8 ± 4.0	0.08 ± 0.75	Unclear
Accelerations of 1.5–1.9 m·s^−2^	2.1 ± 1.5	0.8 ± 1.0	0.12 ± 1.84	Unclear
Accelerations of 2.0–2.4 m·s^−2^	0.2 ± 0.4	0.6 ± 0.7	0.87 ± 1.06	*Likely*
Accelerations ≥ 2.5 m·s^−2^	1.5 ± 1.4	1.9 ± 1.7	0.02 ± 1.27	Unclear
Work:rest ratio	3.6 ± 1.2	2.6 ± 0.9	0.89 ± 0.35	*Almost Certainly*
Player load	91.9 ± 12.9	76.8 ± 13.1	1.20 ± 0.70	*Very Likely*

	SSG_2T_				
Total distance covered (m)	680.7 ± 68.7	683.0 ± 51.2	0.05 ± 0.53	Unclear
Maximum speed (km·h^−1^)	18.2 ± 1.5	19.4 ± 3.0	0.63 ± 0.79	*Likely*
Accelerations of 1.0–1.4 m·s^−2^	4.3 ± 2.8	4.7 ± 3.0	0.07 ± 0.69	Unclear
Accelerations of 1.5–1.9 m·s^−2^	1.3 ± 1.3	0.8 ± 1.3	0.12 ± 4.6	Unclear
Accelerations of 2.0–2.4 m·s^−2^	0.6 ± 0.7	0.7 ± 0.5	0.60 ± 1.74	Unclear
Accelerations ≥ 2.5 m·s^−2^	1.3 ± 1.4	1.4 ± 1.2	0.00 ± 1.46	Unclear
Work:rest ratio	3.1 ± 1.1	2.9 ± 0.7	0.00 ± 0.49	Unlikely
Player load	83.6 ± 13.2	82.6 ± 11.5	0.06 ± 0.45	Unclear

ES: effect size; CL: confidence limits.
